# P-2324. Detections of Endemic Coronaviruses in Medically Attended Respiratory Illness: Clinical Characteristics and Seasonal Trends from Two Public Health Surveillance Studies in Minnesota

**DOI:** 10.1093/ofid/ofae631.2476

**Published:** 2025-01-29

**Authors:** Kristen Bastug, Karen Martin, Kathryn Como-Sabetti, Hannah Friedlander, Jeffrey Sanders, Izzy Brandstetter-Figueroa, Sarah Bistodeau, Anna K Strain, Sara Vetter, Ruth Lynfield, Beth K Thielen

**Affiliations:** University of Minnesota, Minneapolis, Minnesota; Minnesota Department of Health, Saint Paul, Minnesota; Minnesota Department of Health, Saint Paul, Minnesota; Minnesota Department of Health, Saint Paul, Minnesota; Minnesota Department of Health, Saint Paul, Minnesota; University of Minnesota, Minneapolis, Minnesota; Minnesota Department of Health, Saint Paul, Minnesota; Minnesota Department of Health, Saint Paul, Minnesota; Minnesota Department of Health, Saint Paul, Minnesota; Minnesota Department of Health, Saint Paul, Minnesota; University of Minnesota, Minneapolis, Minnesota

## Abstract

**Background:**

Human coronavirus (hCoV) strains OC43, NL63, HKU1, and 229E exist worldwide and have the potential to cause severe disease. Understanding hCoV seasonality, clinical symptoms, and risk factors for severe disease may help predict the impact of emerging respiratory viral pathogens.

**Figure:**
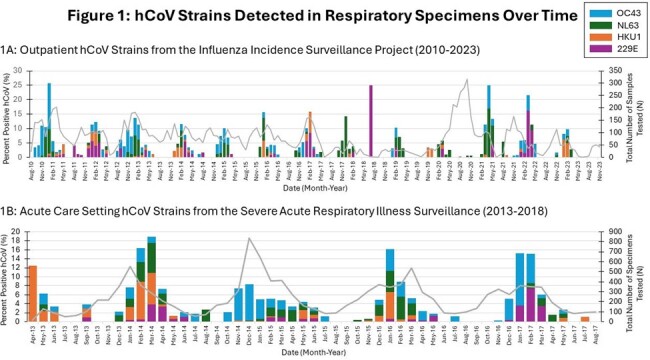

**Methods:**

Eighteen outpatient clinics submitted clinical data and respiratory specimens from patients with influenza-like illness between 2010-2023. Inpatient surveillance data was submitted from 3 hospitals (including one freestanding children’s hospital) between 2013-2018 from patients with acute respiratory illness. Respiratory specimens were tested using a multi-target real-time RT-PCR for 23 bacterial and viral respiratory pathogens.

**Figure:**
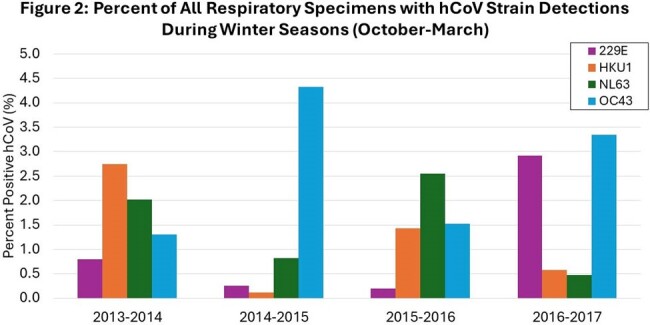

**Results:**

We tested 24,765 specimens (51.2% hospitalized, 48.8% outpatients) and identified 1,207 non-SARS-CoV-2 coronavirus-positive cases (42% hospitalized, 58% outpatient). 74% of inpatient cases were submitted by a standalone children’s hospital. For hCoV+ cases the median outpatient age in years was 36.5 and inpatient age was 0.6. All hCoV strains were detected at higher frequency during the winter months (Figure 1) and sequential winter seasons demonstrated different dominant strains of hCoV (Figure 2). hCoV was co-detected with other viral pathogens in 29% of cases but strains were not co-detected with each other (Figure 3). The median age for cough, diarrhea, fever, URI symptoms, seizure, vomiting and wheeze was ≤1.0 years, while headache, myalgia, and sore throat was 27-31 years (Figure 4). Among hospitalized children with only hCoV detected, comorbidities included neurologic disorder(22%), developmental delay(14%), seizure disorder(14%), cardiovascular disease(13%), prematurity(12%), and asthma(10%). 16% required mechanical ventilation, 27% required ICU admission, and 41% received antiviral therapy. Patients with hCoV+1 other pathogen compared to those with singular hCoV+ infection did not have an increased risk for ICU admission or mechanical ventilation.

**Figure:**
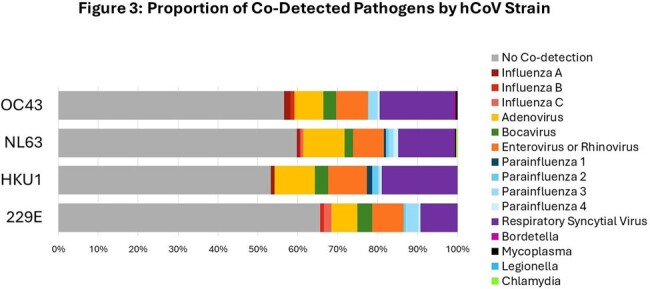

**Conclusion:**

hCoV circulation in Minnesota demonstrates temporal variation in dominant hCoV strain. hCoV infection may present with different symptoms according to the patient’s age. hCoV may contribute to severe respiratory disease in the absence of other pathogenic viruses.

**Figure:**
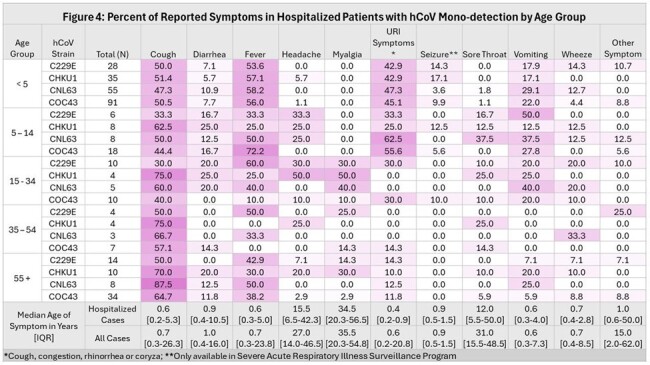

**Disclosures:**

Beth K. Thielen, MD, PhD, GSK: Honoraria|Merck: Grant/Research Support|Sanofi: Honoraria|Seegene: Honoraria

